# Consequences of exercising on ischemia–reperfusion injury in type 2 diabetic Goto-Kakizaki rat hearts: role of the HO/NOS system

**DOI:** 10.1186/s13098-015-0080-x

**Published:** 2015-10-06

**Authors:** Krisztina Kupai, Renáta Szabó, Médea Veszelka, Amin Al Awar, Szilvia Török, Anett Csonka, Zoltán Baráth, Anikó Pósa, Csaba Varga

**Affiliations:** Department of Physiology, Anatomy and Neuroscience, Faculty of Science and Informatics, University of Szeged, Kozep fasor 52, 6726 Szeged, Hungary; Department of Orthodontics and Pediatric Dentistry, Faculty of Dentistry, University of Szeged, 6720 Szeged, Hungary

**Keywords:** Ischemia–reperfusion, Heme oxygenase, Nitric oxide synthase, Exercise

## Abstract

**Background:**

It is well established that physical exercise continues to be one of the most valuable forms of non-pharmacological therapy against diabetes mellitus; however, the precise mechanism remains unknown. The aim of this study was to investigate the cardioprotective effect of voluntary exercise in the Goto-Kakizaki type 2 diabetic rat heart against ischemia–reperfusion injury and to clarify its biochemical background, focusing on the nitric oxide synthase/heme oxygenase system.

**Methods:**

One group of male Goto-Kakizaki rats were allowed voluntary exercise, whereas others were kept sedentary for 6 weeks. At the end of the 6th week the hearts were isolated from both groups and subjected to 45-min coronary occlusion followed by 120-min reperfusion. The infarct size was evaluated by means of triphenyltetrazolium chloride staining. The cardiac and aortic nitric oxide synthase/heme oxygenase activities, plasma leptin and glucose concentrations were also assessed.

**Results:**

The sedentary state prior to the ischemia–reperfusion injury was associated with a significantly higher infarct size (24.56 ± 2.21 vs. 16.66 ± 1.87 %) as compared with that in the voluntary wheel-running group. Exercise altered the constitutive nitric oxide synthase activity; an enhancement was evident in the cardiac (42.5 ± 2.72 vs. 75.6 ± 13.34 pmol/min/mg protein) and aortic tissues (382.5 ± 66.57 vs. 576.9 ± 63.16 pmol/min/mg protein). Exercise lead to a higher heme oxygenase activity (0.68 ± 0.08 vs. 0.92 ± 0.04 nmol bilirubin/h/mg protein) in the diabetic rat hearts. Exercise was associated with lower plasma leptin (192.23 ± 7.22 vs. 169.65 ± 4.6 ng/L) and blood glucose (19.61 ± 0.76 vs. 14.58 ± 0.88 mmol/L) levels.

**Conclusions:**

These results indicate the beneficial role of exercise against myocardial ischemia–reperfusion injury in diabetic rats. These observations in experimental diabetes suggest that the cytoprotective mechanism of exercise involves modulation of the nitric oxide synthase/heme oxygenase system and metabolic parameters that may be responsible for cardioprotection.

**Electronic supplementary material:**

The online version of this article (doi:10.1186/s13098-015-0080-x) contains supplementary material, which is available to authorized users.

## Background

Epidemiological data show that diabetes mellitus (DM) is a major risk factor for cardiovascular diseases, and the mortality from acute myocardial infarction (MI) is 2–6 times higher for DM patients than for non-DM patients. The poor prognosis may be explained at least in part by the diabetic inhibition of endogenous cardioprotective strategies such as pre-and postconditioning against ischemia–reperfusion (IR) injuries in animal models and humans [1]. Establishment a novel strategy to limit the extent of infarction during IR is therefore of great clinical importance.

It has long been known that regular physical activity induces multiple adaptations within the skeletal muscles and the cardiorespiratory system, thereby providing a positive outcome for the prevention or treatment of many metabolic disorders [2]. Moreover, animal studies confirm that regular bouts of aerobic exercise (e.g. running) protect the heart from IR injury [3], while convincing evidence indicates that both short-term (3–5 consecutive days) and long-term (months) endurance exercise (e.g. running) improves the myocardial tolerance to IR injury in both male and female and both young and old animals [4], there is no clear understanding of the cardioprotective effect of exercise in the presence of comorbidities such as DM. To the best of our knowledge, there have been no studies that have evaluated the influence of exercise on the infarct size-limiting effect, and mediators responsible for exercise-induced cardioprotection in rats with spontaneous type 2 DM (T2DM). Therefore, we set out to evaluate the effects of exercie on IR injury, heme oxygenase (HO), and nitric oxide synthase (NOS) in Gioto Kakizaki (GK) rats, undergoing 6 weeks of Voluntary exercise (Wheel-running) followed by acute MI.

## Methods

### Animals

All manipulations were performed in accordance with the standards of the European Community guidelines on the care and use of laboratory animals and had been approved by the Institutional Ethics Committee at the University of Szeged.

**Fig. 1:**
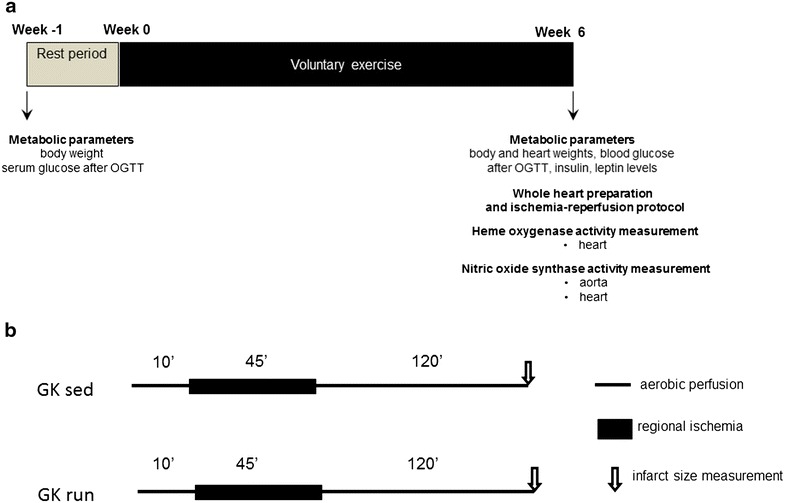
**a** The experimental protocol of the study. The metabolic parameters body weight and serum glucose level after the oral glucose tolerance test (OGTT) were determined. The animals were then allowed to rest for 1 week. At the end of the 6-week exercise or sedentary period, the body and heart weights, and the blood glucose, insulin and leptin levels after the OGTT were measured. The infarct size was determined via the Langendorff perfusion protocols and triphenyltetrazolium chloride staining. At the end of the study, the heme oxygenase and constitutive nitric oxide synthase activities were measured in aorta and heart samples. **b** The experimental Langendorff perfusion protocols. Hearts were subjected to 45-min regional ischemia and 120-min reperfusion after a 10-min stabilization. Infarct size was measured at the end of reperfusion process, via assays with Evans blue and triphenyltatrazolium chloride staining. *GK sed* sedentary Goto-Kakizaki rats, *GK run* voluntary wheel-running Goto-Kakizaki rats

Male GK rats (weighing 270–295 g; Toxi-Coop Zrt., Dunakeszi, Hungary) were randomly assigned to sedentary control (sed, n = 20) and voluntary wheel-running groups (run, n = 20). The running animals were placed individually into cages equipped with a running-wheel (Acellabor Ltd., Budapest, Hungary) and were allowed free access to the wheel for 24 h per day for 6 weeks [5]. The voluntary exercise protocol was selected in an effort to isolate the effects of exercise training from the stress associated with forced training protocols. During the exercise training period, the average running distance was 3.91 ± 1.27 km/day/animal. Control rats were placed in standard holding cages without a running-wheel for the same period. All the animals were housed in a temperature-controlled facility (23 °C) maintained on a 12:12-h light–dark cycle with food and water provided ad libitum. Exercise was started after a 1-week acclimatization period (Fig. 1).

### Whole-heart preparation and IR protocol

To test the hypothesis that exercise is able to provide cardioprotection against IR injury in DM, hearts in each group were exposed to 45-min regional ischemia (occlusion of the left anterior descending artery) and 120-min reperfusion. The rats were deeply anesthetized with diethyl ether, and the hearts were excised, weighed, placed in ice-cold saline, rapidly hung by the aorta on the cannula of a modified Langendorff apparatus and perfused with 37 °C Krebs buffer containing 118 mM NaCl, 4.70 mM KCl, 2.50 mM CaCl_2_, 1.18 mM MgSO_4_, 1.18 mM KH_2_PO_4_, 5.50 mM glucose and 25 mM NaHCO_3_ and gassed with 95 % O_2_-5 % CO_2_ [6].

### Measurement of infarct size

At the end of the 120-min reperfusion, the left anterior coronary artery was religated and the risk zone was labeled with Evans blue dye infused via the aortic root. The hearts were frozen, sectioned transversely from the apex to the base into 5 slices of 2-mm thickness, and incubated in 1 % triphenyltetrazolium chloride for 10 min. After incubation, sections were fixed in 10 % formalin for 10 min, and then placed in phosphate buffer (pH 7.4, 37 °C) for 30 min. Images of the slices were transferred from a digital camera to a personal computer equipped with ImageJ imaging software for analysis.

### Plasma leptin and insulin concentrations

Plasma leptin and insulin concentrations were measured by enzyme-linked immunosorbent assay with a leptin/insulin kit (SunRed Biotechnology Company, Shanghai, China) at the end of the 6-week experiment.

### Oral glucose tolerance test (OGTT)

Measurements were made at the beginning and after 6 weeks of the experiment. After fasting for 12 h, the animals received an oral administration of glucose solution in a dose of 1.0 g/kg body weight. Blood samples were drawn from the tail vein immediately before and 60 and 120 min after glucose administration. Blood glucose concentrations were measured through the use of test reagent strips (Accu Check Active, Roche, Budaörs, Hungary).

### NOS activity

NOS activity was determined by quantifying the conversion of [^14^C]-radiolabeled l-arginine to citrulline by a previously described method with some minor modifications [5]. A whole aorta abdominalis and the whole heart were homogenized as described in connection with the HO activity. The homogenates were centrifuged for 30 min at 20,000*g* at 4 °C. Samples (40 μl) were incubated for 10 min at 37 °C with 100 μL of assay buffer (50 mM KH_2_PO_4_, 1.0 mM MgCl_2_, 50 mM l-valine, 0.2 mM CaCl_2_, 1.0 mM dithiotreitol (DTT), 1.0 mM l-citrulline, 15.5 nM l-arginine, 30 μM flavin adenine dinucleotide, 30 μM flavin mononucleotide, 30 μM tetrahydro-L-biopterin dihydrochloride, 450 μM β-nicotinamide adenine dinucleotide phosphate (β-NADPH), and 12 pM [^14^C]-l-arginine monohydrochloride (all from Sigma-Aldrich, Budapest, Hungary). The reaction was terminated by the addition of 0.5 mL of a 1:1 (v/v) suspension of ice-cold DOWEX (Na^+^ form) in distilled water. The mixture was resuspended with the addition of 850 μL of ice-cold distilled water. The supernatant (970 μL) was removed and radioactivity was determined by scintillation counting. The Ca^2+^ dependence of the NOS activity was determined by the addition of 10 μL of ethylene glycol-bis(β-aminoethyl ether)tetraacetic acid (EGTA; 1 mM, Sigma-Aldrich). NOS activity was confirmed by inhibition with 10 μL of Nω-nitro-l-argininemethyl ester (L-NNA; 3.7 mM, Sigma-Aldrich). The level of inducible NOS (iNOS) was defined as the extent of citrulline formation that was inhibited by L-NNA, but not by EGTA. The constitutive NOS (cNOS) activity was calculated from the difference between the extent of citrulline formation inhibited by EGTA and the total activity. As the nature of the cNOS isoform (eNOS or nNOS) was not determined, this activity is referred to as cNOS. NOS activity was expressed as pmol/min/mg protein.

### Measurement of HO activity in heart tissue

HO activity was measured by measuring bilirubin formation according to Horvath et al. [7]. The whole heart was homogenised (Ultraturrax T25; 13,500 rpm twice for 20 s) in 10 mM N-(2- hydroxyethyl)piperazine-N′-(2-ethanesulfonic acid) (HEPES), 32 mM sucrose, 1 mM DTT, 0.1 mM EDTA, 10 μg/mL soybean trypsin inhibitor, 10 μg/mL leupeptin and 2 ug/mL aprotinin; pH: 7.4. The supernatant was collected by centrifugation for 30 min at 20,000*g* at 4 °C. The reaction mixture contained the following components in a final volume of 1.5 mL: 2 mM glucose 6-phosphate, 0.14 U/mL glucose 6-phosphate dehydrogenase, 15 uM heme, 150 μM β-NADPH, 120 μg/mL rat liver cytosol as a source of biliverdin reductase, 2 mM MgCl_2_, 100 mM potassium phosphate buffer and 150 μL of supernatant. Incubation was carried out in dark at 37 °C for 60 min. The reaction was stopped by putting samples on ice. The bilirubin formed was calculated from the difference between optical densities obtained at 460 and 530 nm. One unit of heme oxygenase activity was defined as the amount of bilirubin (nmol) produced per hour per mg protein.

### Protein determination

Aliquots (20 μL) of the diluted samples (15- or 25-fold with distilled water) were mixed with 980 μL of distilled water, with 200 μL Bradford reagent added to each sample. After mixing and following a 10-min incubation, the samples were assayed spectrophotometrically at 595 nm with a commercial protein assay kit (Bio-Rad Labs, Budapest, Hungary). Protein levels were expressed in units of mg protein/ml.

### Statistical analyses

All data are presented as mean ± SEM. Statistical comparisons were performed with Student’s two-tailed unpaired *t* test. Differences were regarded as significant when the p values were <0.05.

## Results

### Infarct size

**Fig. 2:**
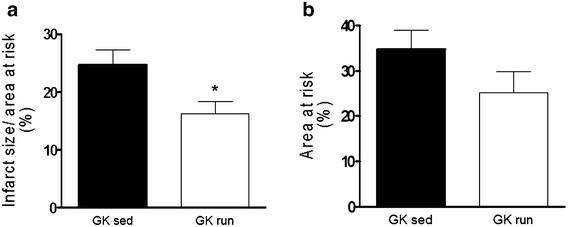
**a** Myocardial infarct size is expressed in % and was determined via triphenyltetrazolium chloride staining. Data are mean ± SEM. Statistical significance: *p < 0.05 relative to the GK sed group. *GK sed* sedentary Goto-Kakizaki rats, *GK run* voluntary wheel-running Goto-Kakizaki rats. **b** Myocardial area at risk is expressed in % after 45-min regional ischemia and 120-min reperfusion. Data are mean ± SEM. *GK sed* sedentary Goto-Kakizaki rats, *GK run* voluntary wheel-running Goto-Kakizaki rats

Sedentary GK hearts subjected to 45-min of regional ischemia and 120-min of reperfusion exhibited an infarct size of 24.56 ± 2.21 %. The voluntary exercise prior to MI resulted in a significantly lower infarct size: 16.66 ± 1.87 % (p < 0.05 vs. GK sed hearts) (Fig. 2a). The areas at risk (AAR) are presented in Fig. 2b. We found no significant difference between voluntary wheel-running and sedentary animals (34.65 ± 4.28 vs. 25.14 ± 4.56 %).

### Body weight and heart weight

There was no significant difference in body weight between the voluntary wheel-running and sedentary GK groups at week-1 (data not shown) or week 0. Throughout the 6-week experimental period, the voluntary exercise animals displayed a lower body weight as compared with the sedentary controls (338 ± 3.7 vs. 314 ± 2.7 g). However, there was no significant difference in heart wet weight between running and sedentary rats (1.58 ± 0.07 vs. 1.61 ± 0.01 g). Data are shown in Additional file 1: Table S1.

### Plasma leptin levels

**Fig. 3:**
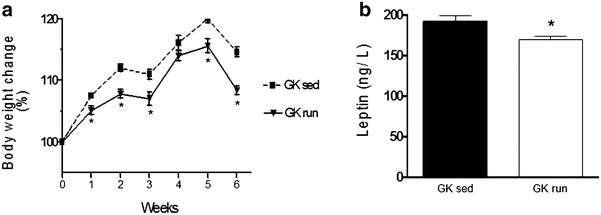
**a** Changes in body weight during the experimental period (expressed in %). Values are shown as mean ± SEM. Statistical significance: *p < 0.05 relative to the GK sed group. *GK sed* sedentary Goto-Kakizaki rats, *GK run* voluntary wheel-running Goto-Kakizaki rats. **b** The plasma leptin levels (expressed in ng/L) at the end of the 6-week exercise. Data are mean ± SEM. Statistical significance: *p < 0.05 relative to the GK sed group. *GK sed* sedentary Goto-Kakizaki rats, *GK run* voluntary wheel-running Goto-Kakizaki rats

The effects of exercise on the plasma leptin level are shown in Fig. 3b. After the 6-week period, the voluntary wheel-running GK rats demonstrated a significantly lower leptin level than that of the sedentary GK rats (192.23 ± 7.22 vs. 169.65 ± 4.6 ng/L).

### OGTT test and insulin levels

**Fig. 4:**
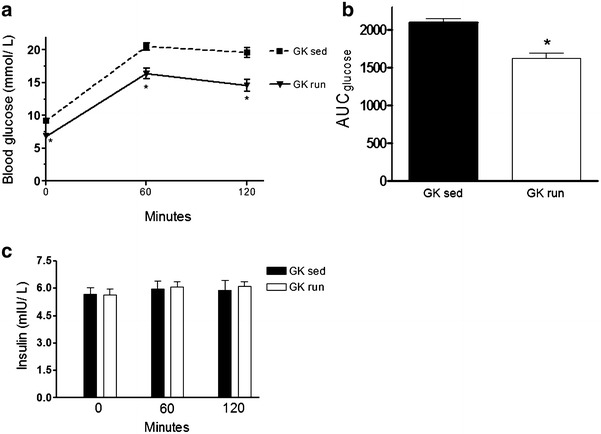
**a** The serum levels of blood glucose (expressed in mmol/L) at the end of the 6-week weeek exercise or sedentary period. Data are mean ± SEM. Statistical significance: *p < 0.05 relative to the GK sed group at the corresponding time. *GK sed* sedentary Goto-Kakizaki rats, *GK run* voluntary wheel-running Goto-Kakizaki rats. **b** The area under the curve (AUC) for glucose after the oral glucose tolerance test at the end of the 6-week exercise or sedentary period. Data are mean ± SEM. Statistical significance: *p < 0.05 relative to the GK sed group. *GK sed* sedentary Goto-Kakizaki rats, *GK run* voluntary wheel-running Goto-Kakizaki rats. **c** The levels of insulin after the oral glucose tolerance test (expresed in mlU/L) at the end of the 6-week exercise or sedentary period. Data are mean ± SEM. *GK sed* sedentary Goto-Kakizaki rats, *GK run* voluntary wheel-running Goto-Kakizaki rats

The changes in glucose sensitivity induced by oral glucose after 6 weeks of exercise are presented in Fig. 4a. The blood glucose concentration peaked at 60 min after glucose administration and then began to decrease. The running GK rats displayed a significantly lower fasting blood glucose level after the 6-week training period. The initial basal glucose level (0 min) was 9.17 ± 0.21 mM/L in the sed group and 6.82 ± 0.3 mM/L in the voluntary running group. Exercise was associated with a significantly lower blood glucose level after 60 (20.51 ± 0.58 vs. 16.39 ± 0.81 mmol/L) and 120 min (19.61 ± 0.76 vs. 14.58 ± 0.88 mmol/L) of the OGTT test. The areas under the curves for glucose (2094.14 ± 53.82 vs. 1625.46 ± 70.11) are depicted in Fig. 4b.

As shown in Fig. 4c, the 6-week exercise did not influence the plasma insulin concentration significantly, though the mean was somewhat higher at 60 and 120 min for the voluntary exercise animals than for the sedentary group.

### Cardiac and aortic cNOS activity

**Fig. 5:**
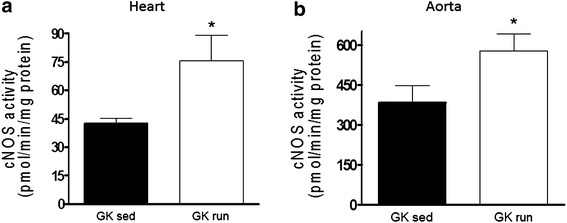
The constitutive nitric oxide synthase (cNOS) activities (expressed in pmol/min/mg protein) in the **a** heart and **b** aorta of GK rats at the end of the 6-week exercise or sedentary period. Data are mean ± SEM. Statistical significance: *p < 0.05 relative to the GK sed group. *GK sed* sedentary Goto-Kakizaki rats, *GK run* voluntary wheel-running Goto-Kakizaki rats

After the 6 weeks of exercise, the cNOS activity was significantly higher both in the heart (42.5 ± 2.72 vs. 75.6 ± 13.34 pmol/min/mg protein) and in the aorta (382.5 ± 66.57 vs. 576.9 ± 63.16 pmol/min/mg protein) relative to the sedentary control group (Fig. 5).

### Cardiac HO activity

**Fig. 6:**
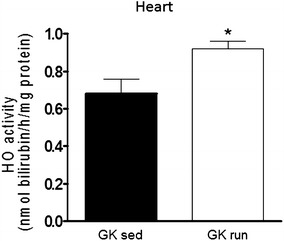
The heme oxygenase (HO) activity in the heart (expressed in nmol bilirubin/h/mg protein) at the end of the exercise or sedentary period. Data are mean ± SEM. Statistical significance: *p < 0.05 relative to the GK sed group. *GK sed* sedentary Goto-Kakizaki rats, *GK run* voluntary wheel-running Goto-Kakizaki rats

The exercise was accompanied by a significantly higher cardiac HO activity (0.68 ± 0.08 nmol bilirubin/h/mg protein) than that in the sedentary group (0.92 ± 0.04 nmol bilirubin/h/mg protein) (Fig. 6).

## Discussion

The aim of the present study was to clarify the effects of voluntary exercise in the T2DM GK rat heart against IR injury, focusing on the NOS/HO system. The main findings of the present study after 6 weeks of voluntary exercise, prior to IR injury (1) attenuation of the infarct size in the T2DM GK heart ex vivo; (2) higher cardiac and aortic cNOS activities; (3) higher cardiac HO activity; and (4) improved metabolic parameters, e.g. plasma leptin and blood glucose concentration.

One limitation of this study is that control Wistar rats were not used. We previously demonstrated that 6 weeks of exercise reduces the myocardial infarct size and improves the angina susceptibility of the heart in Wistar rats [8]. In the current investigation, we focused on the mechanisms in T2DM GK rats.

GK rats are a highly inbred strain derived from outbred, glucose-intolerant Wistar rats that spontaneously develop T2DM within the first few weeks of age [9]. In our study, the basal blood glucose level was significantly lower than that after the OGTT test, demonstrating the impairment of carbohydrate homeostasis. The study revealed that the hyperglycemia in GK rats can be moderated by exercise. Exercise promotes the prevention and treatment of T2DM because of the increase in the capacity of the muscles to capture circulating glucose, due to the decreased intramuscular fat reserves [10].

Experimental animal and human studies suggest that the DM heart is resistant to the cardioprotective effects of pre and postconditioning. We have demonstrated that regular exercise can confer cardioprotection in the T2DM heart. To the best of our knowledge, the present study has provided the first experimental evidence that cardioprotection is induced by voluntary exercise in GK rats.

GK rats at postnatal weeks 6–12 exhibit hyperphagia, hyperglycemia, hyperleptinemia and increased visceral fat accumulation, due to the age-related progression of the islet pathology [11]. Our results indicated that the continuous body weight increase during the 6-week period in the sedentary group, possibly as a result of the insulin resistance, may be reversed by exercise. The average fasting blood glucose and postprandial blood glucose levels in normal Wistar rats are 3.95 ± 1.31 and 5.65 ± 1.63 mmol/L [12]. In the present study, the blood glucose level followed by OGTT was almost 4.5 times higher than in a healthy animal. Both the plasma leptin level and the blood glucose level were markedly lower in the voluntary wheel running GK rats, whereas the plasma insulin levels in the two groups did not differ at the end of the 6th week. In other studies, leptin has been shown to regulate glucose homoeostasis by reversing lipid accumulation and beneficially affecting the insulin resistance and the cell function. Findings in the present study and in previously published studies show that the leptin level declines as a result of the decrease in adipose tissue mass due to chronic exercise [13].

There is general agreement that hyperglycemia and DM lead to an impairment of nitric oxide (NO) production and activity. As a consequence of the high glucose concentration, the quiescence of the endothel cells is lost, the cells acquire new phenotypes, their normal function is impaired, and an “endothel cell dysfunction” is installed. An endothel cell dysfunction is characterized by one or more of the following features: a deficiency in the bioavailability of NO, a reduced level of endothelium-mediated vasorelaxation, hemodynamic downregulation, an impaired fibrinolytic ability, an enhanced turnover, the overproduction of growth factors, the increased expression of adhesion molecules and inflammatory genes, the excessive generation of reactive oxygen species (ROS), an increased level of oxidant stress, and enhanced permeability of the cell layer [14]. Our study showed that 6 weeks of voluntary exercise induced the upregulation of cNOS activity and caused an enhanced level of endothelium- dependent vasorelaxation in GK rats. Although, we did not examine the vascular reactivity in the aorta, previous vascular measurements in DM animals have been reported.

The eNOS activation has recently been linked to the cardioprotective effects of voluntary exercise [15]. In the present study, we confirmed that cNOS is a major player in the mechanism of exercise-induced cardioprotection. Therefore our results are consistent with the findings of previous reports. The blockade of NOS with L-NG-Nitroarginine Methyl Ester reversed NO-mediated vasorelaxation [16]. The blockade of arginase shifted the utilization of arginine from arginase to NOS, resulting in increased NO bioavailability, and normalized the coronary microvascular function in the T2DM GK [17]. Tetrahydrobiopterin (BH4) is an essential cofactor of eNOS [18].

HO catalyzes the oxidation of heme into the biologically active components, biliverdin, carbon monoxide (CO) and iron (ferritin) [19]. Biliverdin, produced in a HO-catalyzed reaction, is reduced to the potent antioxidant bilirubin by the action of biliverdin reductase [20–22]. Bilirubin has been shown to protect isolated perfused rat hearts [23]. CO has been suggested to be cytoprotective due to its potent antifibrinolytic activity [24]. One enzyme system, which has been suggested to interact with HO is the NOS system [25]. NO is synthesized from l-arginine in an enzymatic reaction catalyzed by NOS [26]. Elevated NO levels have been shown to upregulate HO expression, and inhibitors of the HO system inhibit NOS activity [25]. HO activity may also regulate NO generation as NOS could provide heme as a substrate for HO; and HO products can inhibit gene transcription, as demonstrated for bilirubin [27].

The control of eNOS protein levels is a complex operation that is mediated on several levels, including eNOS transcription, mRNA stability and post-translational modifications [28]. In cultured endothelial cells, elevated glucose levels proved to increase the content of eNOS mRNA [29]. Steensberg et al. found that NOS inhibition markedly reduced the exercise-induced expression of HO-1 mRNA in the human skeletal muscle, and infusion of the NO donor nitroglycerine augmented the expression of HO-1 mRNA attenuated by NOS blockade [30]. They therefore suggested that NO is an upstream mediator that controls the exercise-induced expression of HO-1 mRNA expression in the human skeletal muscle.

The process underlying the mechanism of the cardioprotection induced by voluntary exercise needs to be elucidated. In this regard, our current study has suggested that NO and HO upregulation might be of importance for the vascular complications in T2DM GK rat. The regular exercise activated the cNOS system and HO activity.

Furthermore, advanced glycation end-products (AGEs) in the blood seem to be filtered at the glomeruli into the primitive urine, and then absorbed and metabolized at the proximal tubule cells (PTCs) [31]. Exercise markedly accelerated the single nephron glomerular filtration rate of the juxtamedullary nephrons, followed by the accumulation of more AGEs in the PTCs than in a sedentary condition. Accompanying the activation, the PTCs clearly demonstrate the expression of iNOS, which is involved in the resolution of the AGEs [32].

In summary, our results indicate that regular exercise can exert a positive influence on the cardiac and metabolic parameters in T2DM GK rats. 6 weeks of exercise resulted in higher cNOS/HO enzyme activities, lower infarct size, and improved the metabolic parameters relative to those in the sedentary animals. These findings highlight the pivotal role and symbiotic relationship of cNOS/HO in the modulation of the DM phenotype.

## Additional file

10.1186/s13098-015-0080-x Body weight, heart weight and their ratio in the two groups at the end of the 6th week. Results are means ± S.E.M. Statistical significance: *p<0.05 relative to the GK sed group. GK sed = sedentary Goto-Kakizaki rats, GK run = voluntary wheel-running Goto-Kakizaki rats.
