# Supplementary material for: Consequences of exercising on ischemia–reperfusion injury in type 2 diabetic Goto-Kakizaki rat hearts: role of the HO/NOS system
Source: Diabetol Metab Syndr. 2015 Oct 6;7:85. doi: 10.1186/s13098-015-0080-x (PMC4595319; doi:10.1186/s13098-015-0080-x)
Supplement: Supplementary file 1 — 10.1186/s13098-015-0080-x Body weight, heart weight and their ratio in the two groups at the end of the 6th week. Results are means ± S.E.M. Statistical significance: *p<0.05 relative to the GK sed group. GK sed = sedentary Goto-Kakizaki rats, GK run = voluntary wheel-running Goto-Kakizaki rats. [file 13098_2015_80_MOESM1_ESM.docx]

|  | **Sed** | **Run** |
| --- | --- | --- |
| Body weight (g) | 338 ± 3.7 | 314 ± 2.7 * |
| Heart wet weight (g) | 1.583 ± 0.071 | 1.609 ± 0.0092 |
| Heart/body weight ratio | 0.0047 ± 0.0002 | 0.0051 ± 0.0003 |

**Table. S1**

Values are ± SEM; *P<0.05 vs GK sed (n=14-17)

Values were measured at week 6th
